# T2 mapping of the distal sciatic nerve in healthy subjects and patients suffering from lumbar disc herniation with nerve compression

**DOI:** 10.1007/s10334-020-00832-w

**Published:** 2020-02-11

**Authors:** Nico Sollmann, Dominik Weidlich, Elisabeth Klupp, Barbara Cervantes, Carl Ganter, Claus Zimmer, Ernst J. Rummeny, Thomas Baum, Jan S. Kirschke, Dimitrios C. Karampinos

**Affiliations:** 1 Department of Diagnostic and Interventional Neuroradiology, Klinikum rechts der Isar, Technische Universität München, Munich, Germany; 2 TUM-Neuroimaging Center, Klinikum rechts der Isar, Technische Universität München, Munich, Germany; 3 Department of Diagnostic and Interventional Radiology, Klinikum rechts der Isar, Technische Universität München, Munich, Germany

**Keywords:** Magnetic resonance spectroscopy, Peripheral nervous system diseases, Sciatica, Spine

## Abstract

**Objective:**

To measure T2 values for magnetic resonance neurography (MRN) of the healthy distal sciatic nerve and compare those to T2 changes in patients with nerve compression.

**Materials and methods:**

Twenty-one healthy subjects and five patients with sciatica due to disc herniation underwent MRN using a T2-prepared turbo spin echo (TSE) sequence of the distal sciatic nerve bilaterally. Six and one of those healthy subjects further underwent a commonly used multi-echo spin-echo (MESE) sequence and magnetic resonance spectroscopy (MRS), respectively.

**Results:**

T2 values derived from the T2-prepared TSE sequence were 44.6 ± 3.0 ms (left) and 44.5 ± 2.6 ms (right) in healthy subjects and showed good inter-reader reliability. In patients, T2 values of 61.5 ± 6.2 ms (affected side) versus 43.3 ± 2.4 ms (unaffected side) were obtained. T2 values of MRS were in good agreement with measurements from the T2-prepared TSE, but not with those of the MESE sequence.

**Discussion:**

A T2-prepared TSE sequence enables precise determination of T2 values of the distal sciatic nerve in agreement with MRS. A MESE sequence tends to overestimate nerve T2 compared to T2 from MRS due to the influence of residual fat on T2 quantification. Our approach may enable to quantitatively assess direct nerve affection related to nerve compression.

## Introduction

Magnetic resonance neurography (MRN) of the peripheral nervous system (PNS), the application of magnetic resonance imaging (MRI) for the depiction and study of peripheral nerves of the human body, is increasingly applied in vivo. Advances in protocols and MRI scanner techniques now allow depicting even small or highly oblique nerves originating from the brachial plexus or lumbosacral plexus (LSP) [1–3]. A prominent nerve originating from the LSP is the sciatic nerve, which has been routinely under investigation in studies using MRN in the past. It is composed of tibial and common peroneal components of sciatic plexus fibers (rami of the L4 to S3 nerve roots) that become enclosed in a common nerve sheath [4]. The sciatic nerve enters the gluteal region and courses inferiorly between the adductor magnus and gluteus maximus muscles to the distal one-third of the thigh where it divides into the tibial and common peroneal trunks [4].

Using qualitative MRN and structural evaluations, the sciatic nerve has shown to be affected in various pathologies, such as hamstring injury due to different causes [5], injection injury after iatrogenic intramuscular infiltrations [6], injury related to hip replacement surgeries [7], perineuriomas [8], Fabry disease [9–11], hereditary motor and sensory neuropathies such as Charcot–Marie–Tooth disease (CMTD) [12], or diabetic and other neuropathies [13, 14]. These studies conducted primarily visual inspections of the sciatic nerves to detect changes in signal intensities, discontinuities, or lesion loads, often together with evaluations of morphological nerve sizes and volumes [5–14].

In addition to such qualitative MRN, (semi-)quantitative approaches have been introduced recently. Diffusion parameters derived from diffusion tensor imaging (DTI) have been evaluated for the sciatic nerve in patients with muscular disorders such as myotonic dystrophy [15], radiculopathy [16], chronic inflammatory demyelinating polyneuropathy (CIDP) [17], CMTD [18], oxaliplatin-induced polyneuropathy [19], or diabetic neuropathy [20, 21]; however, studies have not always been successful in showing changes in diffusion parameters related to the respective pathologic conditions. In addition to that, a study relating semi-quantitatively measured magnetic transfer ratios to clinical markers found interesting correlations in patients with CMTD [22]. Further, measurements of T2 values have been achieved in addition in the same patients or diseases [18–21, 23], and also in patients suffering from multiple sclerosis (MS) [24] or amyloid polyneuropathy [25]. In general, nerves enclose multiple water components with distinct relaxation times, resulting in a multi-compartment T2 signal decay [26–28]. Most studies report one short T2 component (around 20 ms) and two or three longer T2 components (50–200 ms). To address these complex relaxation properties of nerve tissue, the signal decay curve can be either sampled densely with the signal decay being fully modelled, or the respective echo times (TEs) of the imaging sequence are selected in such a way that certain compartments are not visible on the signal decay curve.

Despite being increasingly used and yielding promising results among patients with primarily neuropathic diseases, evidence is still lacking for even more common conditions such as sciatica occurring due to structural pathology such as degenerative disc disease with nerve compression [29, 30]. Quantitative MRN by means of measuring T2 values is still at an incipient stage and mainly restricted to specialized centers. Hence, reference data regarding T2 values of the sciatic nerve and investigations of reliability are largely lacking. To date, the report of normal values regarding T2 of the sciatic nerve is restricted to few data of control groups of healthy or asymptomatic patients formed for comparisons to patients with diseases and focal or systemic PNS affections. A recent study explicitly focused on the assessment of normal T2 values of the sciatic nerve by investigating a cohort of 60 healthy individuals by means of MRN using a multi-echo spin-echo (MESE) sequence for T2 relaxometry at 3 Tesla [31]. This study reported on physiological T2 values in five men and five women of every decade between 20 and 80 years and additionally evaluated associations with demographic variables [31]. However, the issue of reliability has not been explicitly addressed, and it remains unclear whether the sequence used is optimal for realistic assessment of T2 values. Hence, further data on physiological T2 values and systematic evaluations of reliability are highly needed for a more profound understanding of T2 values of the sciatic nerve, thus paving the way to identify valid biomarkers in quantitative MRN of the PNS.

Against this background, the objective of the present study is (1) to provide T2 values for the healthy distal sciatic nerve and to evaluate reliability of quantitative MRN by T2 mapping in healthy subjects, (2) to compare our novel T2 mapping approach against T2 measurements in a commonly used MESE sequence (having nerve-water T2 from MRS as the reference standard), and (3) to evaluate potential alterations in T2 values of the distal sciatic nerve due to degenerative disc disease with nerve compression at the level of the LSP in patients suffering from unilateral sciatica. We hypothesize that T2 mapping using a T2-prepared turbo spin echo (TSE) sequence shows good agreement with nerve-water T2 from MRS, due to robust suppression of residual fat that can affect T2 quantification whilst being characterized by high precision. Furthermore, we propose that patients with nerve compression at the level of the LSP present with unilaterally elevated T2 values of the distal sciatic nerve when compared to the unaffected side or healthy subjects, probably due to edematous changes or swelling of nerves related to proximal compressive effects.

## Materials and methods

### Ethics

The study protocol was approved by the local ethics commission (registration number 408/15S) and was followed in accordance with the Declaration of Helsinki. Written informed consent was obtained from all included subjects.

### Study cohort

This prospective study enrolled twenty-one healthy volunteers (14 males and 7 females, mean age 28.0 ± 3.7 years, age range 22–39 years) and five patients (3 males and 2 females, mean age 68.1 ± 11.2 years, age range 48–75 years) with unilateral sciatica due to degenerative disc disease. Inclusion criteria were (1) written informed consent, (2) age above 18 years, (3) strict unilateral sciatica matching at least one dermatome with the presence of symptoms (pain with or without additional paresthesia or impairment of motor function) for at least 4 weeks and not longer than 6 months (only for the patient group), and (4) previous anatomical MRI suggesting degenerative disc disease with disc herniation and unilateral nerve contact of degenerated disc material (only for the patient group). Exclusion criteria for both the healthy volunteers and patients were (1) pregnancy, (2) contraindications for MRI, and (3) previous surgery at the hip, thigh, or lumbar spine (except for surgical decompression without any implants).

The healthy volunteers and patients underwent MRN during a 1-day study visit. Furthermore, the patients underwent detailed clinical examinations that included assessments of coordination, sensory function, and muscle strength according to the British Medical Research Council (BMRC) scale. Additionally, the Oswestry Disability Index (ODI) was calculated [32, 33], and rating of sciatica according to a visual analogue scale (VAS) was obtained [34, 35].

### Acquisition of magnetic resonance imaging

MRN was performed using a whole-body 3-Tesla MRI scanner (Ingenia, Philips Healthcare, Best, The Netherlands) using a 16-channel T/R knee coil, with the lower edge of the coil ending at the upper edge of the patella, thus covering the middle to lower thigh region. Both thighs were scanned subsequently, with the side scanned first being subject to randomization. The scanning protocol included the following sequences:

- Two-dimensional (2D) high-resolution T2-weighted DIXON TSE sequence (acquired in all healthy volunteers and patients): field of view (FOV) = 140 × 150 × 104 mm^3^, acquisition voxel size = 0.3 × 0.3 × 8 mm^3^, TE = 60 ms, repetition time (TR) = 2500 ms, scan time = 6 min 10 s, SENSE acceleration factor = 2.0.
- Three-dimensional (3D) T2-prepared TSE sequence (acquired in all healthy volunteers and patients): FOV = 140 × 150 × 52 mm^3^, acquisition voxel size = 0.7 × 0.7 × 8 mm^3^, TE = 30 ms, TR = 1600 ms, scan time = 3 min 30 s, fat suppression using Spectral Attenuated Inversion Recovery (SPAIR), SENSE acceleration factor = 2.5, T2 preparation applying a B1-insensitive rotation radiofrequency pulse as previously used [36, 37], T2 preparation duration = 20/40/60/80 ms. Together with the effective T2 weighting during the TSE readout, the effective TE of the sequence was 47.9 ms, 67.9 ms, 87.9 ms, and 107.9 ms.
- 2D MESE sequence (acquired in six healthy volunteers): FOV = 160 × 160 × 38 mm^3^, acquisition voxel size = 0.9 × 0.8 × 3.5 mm^3^, 12 echoes with TE_1_/ΔTE = 10 ms/10 ms, TR = 2400 ms, scan time = 6 min 48 s, fat suppression using SPAIR, SENSE acceleration factor = 2.4. For this sequence, the standard vendor’s implementation was employed.
- Stimulated Echo Acquisition Mode (STEAM) magnetic resonance spectroscopy (MRS; acquired in one healthy volunteer): volume of interest (VOI) = 5 × 5 × 10 mm^3^, mixing time (TM) = 11 ms, TE = 20/30/40/50/60/70/80/90/100/110/120 ms, TR = 4000 ms, scan time = 6 min 30 s, eight averages. The planning of the MRS sequence was based on the T2-weighted DIXON images and the VOI was placed in such a way that it incorporated the distal sciatic nerve.

### Data processing and measurements of T2 values

#### T2 mapping

**Fig. 1:**
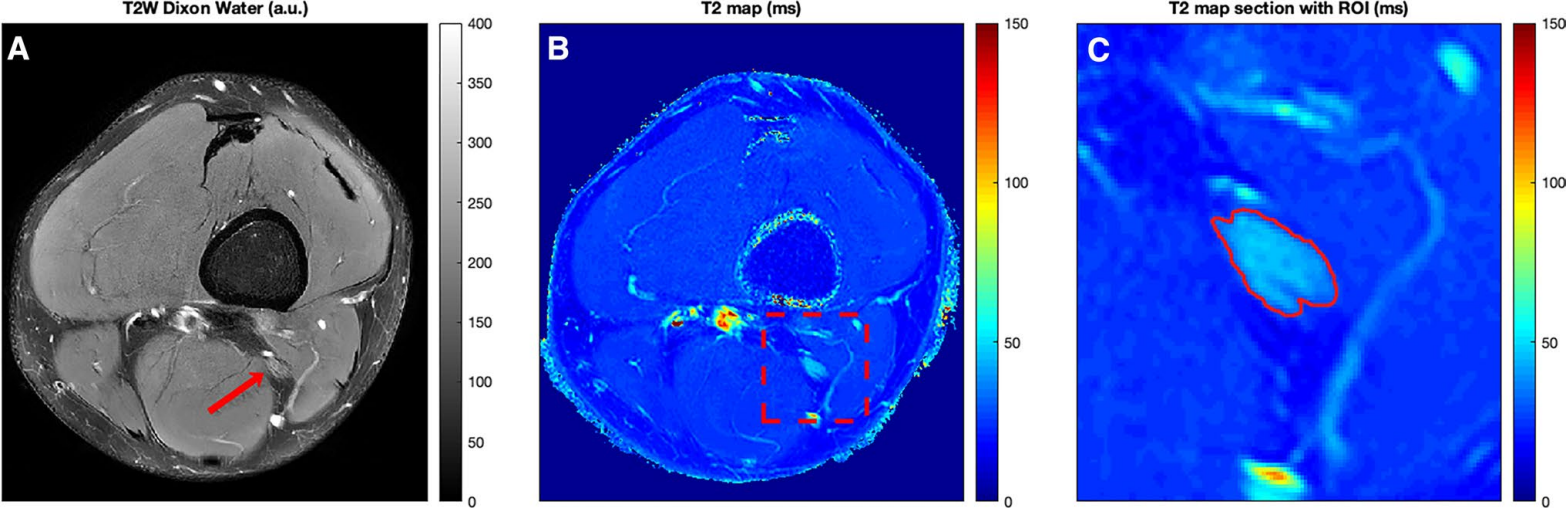
T2 mapping of the distal sciatic nerve and placement of the region of interest (ROI). Representative axial slices of the two-dimensional (2D) T2-weighted DIXON turbo spin echo (TSE) sequence and the T2 map derived from the three-dimensional (3D) T2-prepared TSE sequence in a healthy volunteer (lower thigh region of the left leg). The red arrow points to the lower sciatic nerve in the 2D T2-weighted DIXON TSE sequence (**a**), the red square encloses the nerve in the T2 map (**b**). Polygonal ROIs were placed in the three consecutive axial slices depicting the distal sciatic nerve, with ROIs contouring the whole sciatic nerve caliber without the inclusion of surrounding soft tissue or blood vessels (**c**). The 2D T2-weighted DIXON TSE sequence is scaled with arbitrary units (a.u.), T2 values were extracted in ms. The same approach of ROI placement was followed for the measurements of T2 values in T2 maps derived from a 2D multi-echo spin-echo (MESE) sequence

**Fig. 2:**
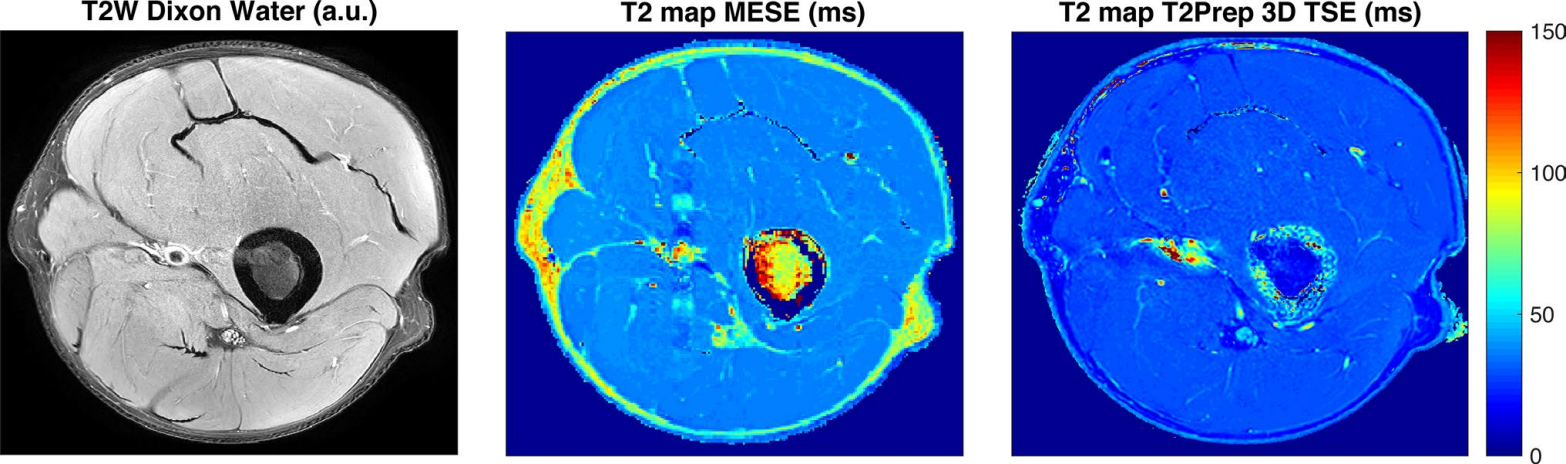
Comparison of T2 maps of the distal sciatic nerve. Comparison between the two-dimensional (2D) T2-weighted DIXON turbo spin echo (TSE) sequence [scaled with arbitrary units (a.u.)], the T2 map derived from the 2D multi-echo spin-echo (MESE) sequence (T2 map MESE), and the T2 map derived from the three-dimensional (3D) T2-prepared TSE sequence (T2 map T2Prep 3D TSE) in a healthy volunteer (lower thigh region of the left leg). Note the higher T2 values (in ms) in the T2 maps of the 2D MESE sequence for structures with fat (particularly the thigh musculature) in comparison to the T2 maps of the 3D T2-prepared TSE sequence

After exporting the data from the MRI scanner, T2 values derived from the 3D T2-prepared TSE sequence (in all healthy volunteers and all patients) and from the 2D MESE sequence (in six healthy volunteers) were calculated offline on a voxel-by-voxel basis with a combination of variable projection and golden section search assuming a mono-exponential signal decay [38–41]. A mono-exponential signal decay of the reported multi-compartment T2 decay of nerve tissue was employed because of the inherently strong T2 weighting of the proposed 3D T2-prepared TSE sequence. The resulting T2 maps (one T2 map for the left and right side per healthy subject or patient and sequence) were successively opened in Horos (version 1.1.7; https://www.horosproject.org) and color-coded according to a standardized scheme (Figs. 1,2) [40, 41].

First, the distal sciatic nerve was carefully identified on axial slices of the color-coded T2 maps, co-registered to uncolored anatomical images of the 2D T2-weighted DIXON TSE sequence that served as reference for enhanced structural interpretability. Then polygonal regions of interest (ROIs) were manually placed in the three consecutive slices directly above the nerve bifurcation of the sciatic nerve, thus above the split into tibial and common peroneal trunks (Figs. 1, 2) [4]. Each of these ROIs contoured the whole nerve caliber whilst inclusion of surrounding soft tissue or blood vessels was avoided. T2 values were separately extracted from the three consecutive ROIs drawn in the T2 maps derived from the 3D T2-prepared TSE sequence and the 2D MESE sequence, if available (Figs. 1,2). The approach of ROI drawing was the same in healthy subjects and patients and for both sides, respectively.

In healthy volunteers, the described approach of ROI positioning and extraction of T2 values from T2 maps of the 3D T2-prepared TSE sequence and 2D MESE sequence was performed by a medical doctor [with experience in neuroradiological imaging since 2012, reader 1 (R1)]. Furthermore, to assess inter-reader reliability, ROI positioning and extraction of T2 values from T2 maps of the 3D T2-prepared TSE sequence was also performed by a second medical doctor [with experience in neuroradiological imaging since 2014, reader 2 (R2)]. In patients, only R1 evaluated the imaging data of both sides using the T2 maps of the 3D T2-prepared TSE sequence. During analyses, readers were strictly blinded to the measurements of each other, and R1 was furthermore blinded to the side of sciatica in patients and to previous anatomical MRI that suggested degenerative disc disease with disc herniation.

#### Spectroscopy

The processing of the MRS data was performed with in-house software and included zero-order phasing, Gaussian apodization, and frequency alignment of single acquisitions. Peak area quantification was achieved considering eight fat peaks and was considered separately for each TE. The T2 fitting was only performed on the water peak signal and was carried out twice, once considering all available TEs and once only considering the matched TEs with the 3D T2-prepared TSE sequence.

### Statistical analyses

Statistics and generation of graphs were performed using GraphPad Prism (version 6.0; GraphPad Software Inc., La Jolla, CA, USA) and SPSS (version 23.0; IBM, Chicago, IL, USA). A *p* value < 0.05 was considered statistically significant.

In healthy volunteers, the measured T2 values of the ROIs of the three consecutive slices were averaged, which was achieved separately for both sides, the measurements of both readers, and for values of the T2 maps as derived from the 3D T2-prepared TSE sequence or 2D MESE sequence. Differences between sides were assessed using Wilcoxon matched-pairs signed rank tests, which was achieved separately for the measurements of both readers and measurements derived from the T2 maps of the 3D T2-prepared TSE sequence and the 2D MESE sequence. Moreover, Wilcoxon matched-pairs signed rank tests were conducted to compare T2 values of the 3D T2-prepared TSE sequence and the 2D MESE sequence. To assess inter-reader reliability for T2 mapping derived from the 3D T2-prepared TSE sequence, we calculated the root mean square error (RMSE) and the root mean square coefficient of variation (RMSCV) based on the values obtained from R1 and R2, again separately considering T2 values of the left and right sides [42]. Since MRS was only acquired in one healthy subject, no statistical testing was performed for related T2 values.

In patients, the measured T2 values by R1 derived from the ROIs of the three consecutive slices were averaged, which was achieved separately for the affected side and the contralateral side with respect to the side of disc herniation and unilateral nerve contact. The T2 values of the affected side were compared to the left-sided and right-sided values obtained from the healthy subjects derived from evaluations of R1 according to T2 mapping from the 3D T2-prepared TSE sequence using Mann–Whitney tests.

## Results

### Healthy subjects

MRN including T2 mapping of the distal sciatic nerve with the 3D T2-prepared TSE sequence was successfully achieved bilaterally in all enrolled healthy volunteers, with the distal sciatic nerve being clearly interpretable in all subjects. Thus, forty-two T2 values (twenty-one T2 values per side as the result of averaging measurements obtained from ROIs in three consecutive slices) derived from the 3D T2-prepared TSE sequence were available for analyses. Furthermore, six volunteers also underwent bilateral scanning of the distal sciatic nerve with the 2D MESE sequence, leading to twelve T2 values (six T2 values per side as the result of averaging measurements obtained from ROIs in three consecutive slices) derived from this sequence. One volunteer also underwent MRS bilaterally using STEAM.

#### T2 mapping—3D T2-prepared TSE sequence

**Fig. 3:**
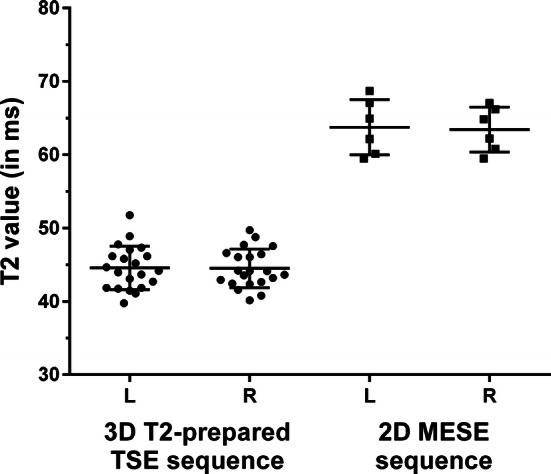
T2 values of the distal sciatic nerve. Scatter dot plots with lines indicating means and standard deviation for T2 values (in ms) of the distal sciatic nerve as measured in T2 maps derived from the three-dimensional (3D) T2-prepared turbo spin echo (TSE) sequence and the two-dimensional (2D) multi-echo spin-echo (MESE) sequence. Respective values are separately shown for the left (L) and right (R) distal sciatic nerves

The T2 values derived from the 3D T2-prepared TSE sequence were 44.6 ± 3.0 ms (R1) and 45.7 ± 3.3 ms (R2) for the left and 44.5 ± 2.6 ms (R1) and 45.7 ± 3.0 ms (R2) for the right distal sciatic nerve (Table 1, Fig. 3). There was no statistically significant difference between sides (R1 *p* = 0.87, R2 *p* = 0.92; Table 1, Fig. 3). Furthermore, there was high inter-reader agreement regarding measurements of T2 values (Table 1).

**Table 1:** T2 values and inter-reader reliability

		R1	R2	RMSE	RMSCV
T2 value (in ms; mean ± standard deviation)					
Left side		44.6 ± 3.0	45.7 ± 3.3	0.29	0.67%
Right side		44.5 ± 2.6	45.7 ± 3.0	0.34	0.73%
*p* value		0.87	0.92		

T2 values (in ms) as mean ± standard deviation as measured in T2 maps of the three-dimensional (3D) T2-prepared turbo spin echo (TSE) sequence for the left- and right-sided distal sciatic nerves of twenty-one healthy subjects are depicted. Measurements were performed by two readers (R1 and R2), with evaluations of inter-reader reliability presented as root mean square error (RMSE) and root mean square coefficient of variation (RMSCV). There were no statistically significant differences in T2 values between sides among the healthy subjects according to the evaluations of both readers (*p* > 0.05)

**Fig. 4:**
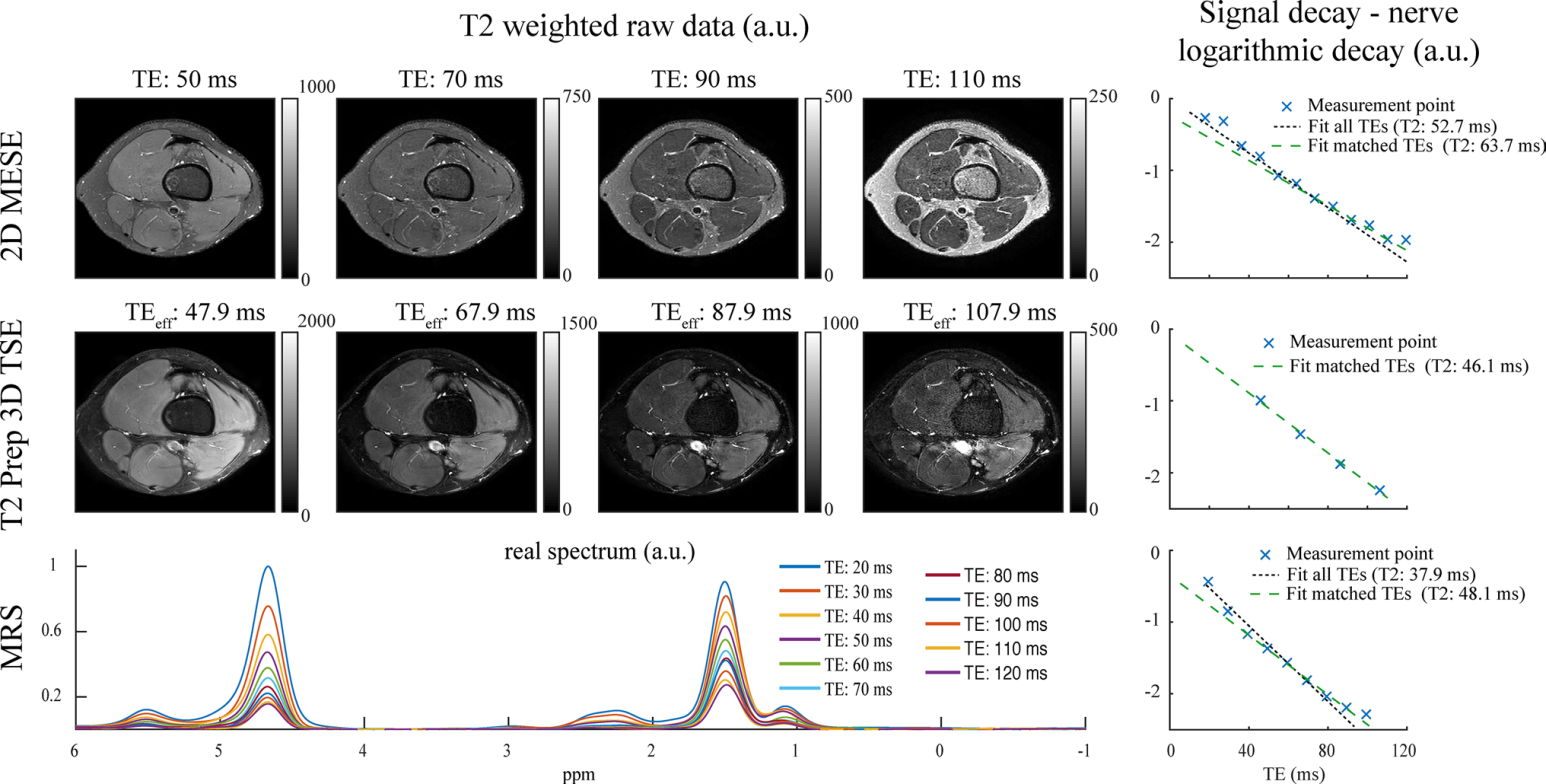
T2-weighted raw images and nerve signal decay curves. Comparison of the raw T2-weighted images [scaled with arbitrary units (a.u.)] for the two-dimensional (2D) multi-echo spin-echo (MESE) sequence (upper row) and the three-dimensional (3D) T2-prepared turbo spin echo (TSE) sequence (middle row) in a healthy volunteer (lower thigh region of the left leg). For the 2D MESE sequence, the echo times (TEs) that approximately match the effective T2 weighting times of the 3D T2-prepared TSE sequence are shown. The bottom row shows the acquired spectrum of the distal sciatic nerve (scaled with a.u.). In the right column, the nerve signal decay is shown for the three sequences with the corresponding T2 fit incorporating all available TEs (black dotted line) or matched TEs (green dashed line). When the TEs are matched, a good agreement between magnetic resonance spectroscopy (MRS) and the 3D T2-prepared TSE sequence can be seen, whereas the T2 in the 2D MESE sequence is elevated regardless of TE selection

Figure 4 shows the T2-weighted raw images of the 3D T2-prepared TSE sequence and the corresponding nerve signal decay curves. A homogenous and consistent fat suppression can be observed for all effective TEs. The nerve signal decay shows a mono-exponential course.

#### T2 mapping—2D MESE sequence

The T2 values derived from the 2D MESE sequence were 63.7 ± 3.8 ms for the left and 63.4 ± 3.1 ms for the right distal sciatic nerve (R1; Fig. 3). There was no statistically significant difference between sides (R1 *p* = 0.56; Fig. 3). Thus, in comparison to T2 values derived from the 3D T2-prepared TSE sequence, T2 values of the 2D MESE sequence were clearly higher for both the left and right sides (R1 *p* = 0.03 each; Fig. 3).

Figure 4 shows the T2-weighted raw images of the 2D MESE sequence for the TEs matching the effective TEs of the 3D T2-prepared TSE sequence. The signal of the fat is increasing with increasing TEs. In the corresponding signal decay curves, stimulated echoes and an increasing fat component at longer TEs are observed. The T2 value obtained by fitting all TEs and by fitting the matched TEs is elevated compared to the values derived from the 3D T2-prepared TSE and MRS sequences.

#### Spectroscopy

The T2 values derived from MRS were 48.1 ms (37.9 ms unmatched) for the left and 43.6 ms (40.7 ms unmatched) for the right distal sciatic nerve for the volunteer examined (R1).

Figure 4 shows the comparison of MRS with the results from the T2 mapping derived from the 3D T2-prepared TSE sequence and the 2D MESE sequence, respectively. The signal decay appears to be multi-compartmental. However, at longer TEs (corresponding to the effective TEs of the 3D T2-prepared TSE sequence), a mono-exponential decay can be observed. The T2 values obtained by the 2D MESE sequence are considerably higher compared to the T2 values derived from the 3D T2-prepared TSE sequence and when compared to MRS measurements when the signal decay is fitted for the matched TE range.

### Patients

**Fig. 5:**
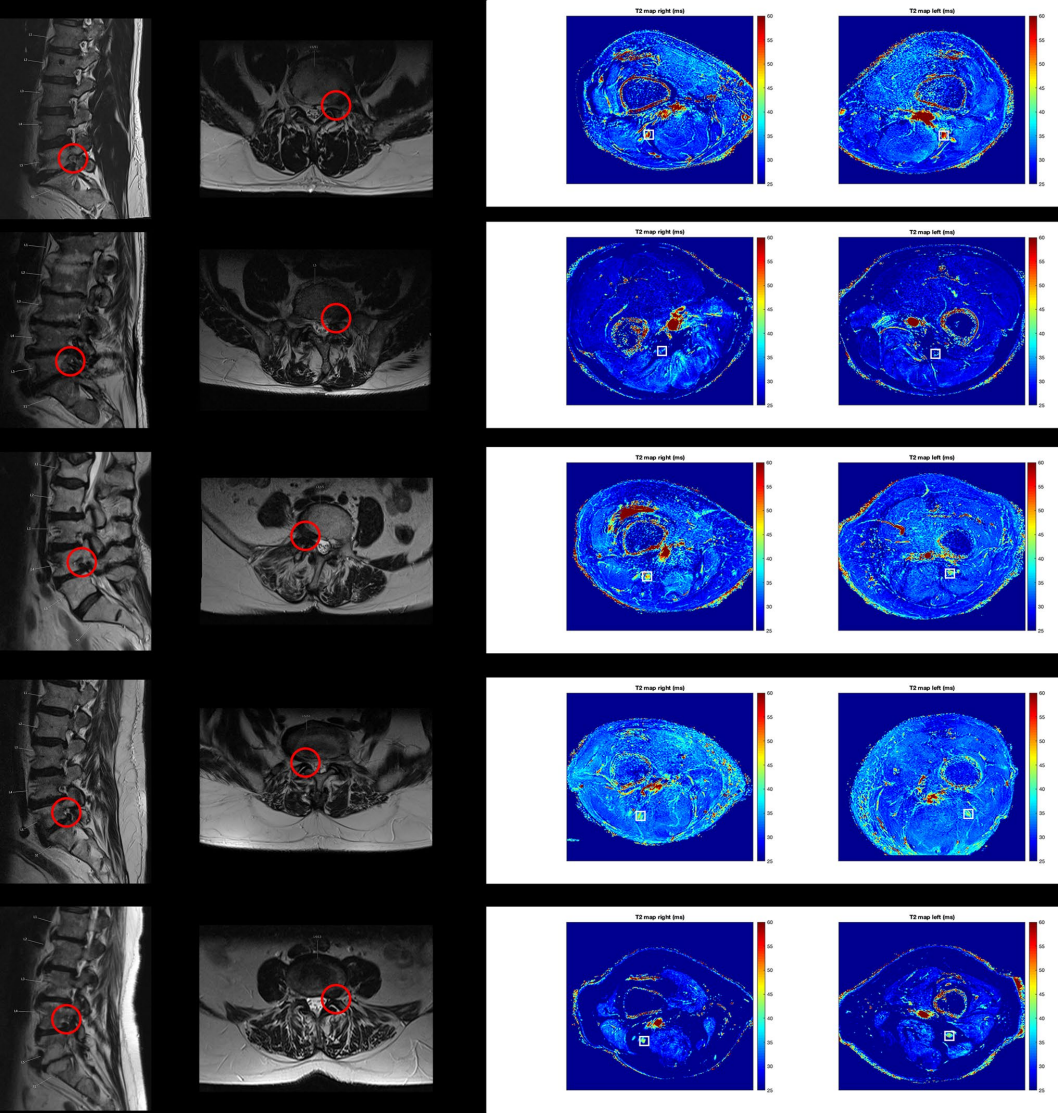
Case series of patients. Sagittal and axial slices of structural magnetic resonance imaging (MRI) from a case series of five patients with unilateral sciatica due to degenerative disc disease with disc herniation and unilateral nerve contact of degenerated disc material (red circles). Furthermore, representative axial T2 maps derived from the three-dimensional (3D) T2-prepared turbo spin echo (TSE) sequence are shown for the right and left lower thigh regions, depicting the distal sciatic nerve (white squares). Mean ± standard deviation of measured T2 values of the lower sciatic nerve was 61.5 ± 6.2 ms for the affected side and 43.3 ± 2.4 ms for the contralateral, unaffected side

Five patients underwent MRN with T2 mapping of the distal sciatic nerve bilaterally using the 3D T2-prepared TSE sequence. These patients initially presented in the outpatient clinic with unilateral sciatica due to degenerative disc disease with disc herniation and unilateral nerve contact of degenerated disc material according to previous anatomical MRI (Fig. 5). Neuroforaminal to extra-neuroforaminal disc herniations with nerve contact affecting L4 or L5 were present in these patients (Fig. 5). A mean ODI score of 35.2 ± 10.7 was registered, with a mean pain rating according to the VAS of 7.4 ± 1.1 points. None of the patients suffered from a manifest hemiparesis according to the BMRC scale. Two patients reported previous surgery for decompression at a different level than the currently affected level 37 and 17 years ago, respectively.

When discriminating between T2 values of the affected and non-affected side derived from the 3D T2-prepared TSE sequence for the distal sciatic nerve, patients showed T2 values of 61.5 ± 6.2 ms for the affected side and 43.3 ± 2.4 ms for the contralateral, unaffected side (R1). When comparing the T2 values of the affected side against the T2 values obtained in the healthy subjects, significant differences were observed for both the comparison against left-sided and right-sided normal T2 values (R1 *p* < 0.0001 each).

## Discussion

We successfully applied MRN with T2 mapping in a cohort of twenty-one healthy subjects, reporting on normal T2 values for the distal sciatic nerve of approximately 45 ms at 3 Tesla when considering a 3D T2-prepared TSE sequence. A more commonly used 2D MESE sequence showed higher T2 values for the distal sciatic nerve of around 63 ms; however, data derived from MRS were in accordance with the T2 values derived from the 3D T2-prepared TSE sequence. Patients showed significantly elevated T2 values unilaterally for the distal sciatic nerve when compared to healthy subjects applying the 3D T2-prepared TSE sequence.

The sciatic nerve has regularly been under investigation by means of MRN. However, until recently, primarily qualitative MRN has been carried out, with the signal of the sciatic nerve or its morphology being altered in the context of different pathologic conditions such as traumatic or iatrogenic injury and tumors such as perineuriomas [5–8], Fabry disease [9–11], CMTD [12], or diabetic and other neuropathies [13, 14]. Importantly, (semi-)quantitative MRN might be more objective and potentially more robust and reproducible when compared to mere qualitative MRN, with DTI, for instance, being capable of contributing with quantitatively assessable changes by means of diffusion parameters that have already been evaluated among patients with muscular disorders such as myotonic dystrophy [15], radiculopathy [16], CIDP [17], CMTD [18], or different kinds of neuropathies [19–21]. Further, measurements of T2 values have been obtained recently in the same diseases and further chronic systemic diseases, such as MS or amyloid polyneuropathy [18–21, 23–25]. Although the approach of (semi-)quantitative MRN using DTI or evaluations of T2 is on the increase, normal values from healthy subjects and analyses of reliability are still largely missing, thus hampering clear identification of pathologic values due to the lack of references.

To the authors’ knowledge, normal T2 values of the sciatic nerve have been primarily gathered from few control groups of healthy subjects or asymptomatic patients that were formed for comparisons to diseased cohorts with symptoms, with only one recent study being available to date that aimed on the distinct exploration of normal T2 values of the sciatic nerve [31]. This study investigated 60 healthy individuals by applying a MESE sequence for T2 relaxometry at 3 Tesla and demonstrated physiological T2 values in 5 men and 5 women of every decade between 20 and 80 years [31]. The study reported mean T2 values of 70.85 ± 4.91 ms [31]. These values are, however, clearly higher in comparison to the T2 values obtained with the 3D T2-prepared TSE sequence in the present study, which were 44.6 ± 3.0 ms for the left and 44.5 ± 2.6 ms for the right distal sciatic nerve (according to measurements of R1). The 2D MESE sequence, which was conducted in addition to the 3D T2-prepared TSE sequence for T2 measurements in six subjects, showed T2 values that were comparable to those reported in the previous study, accounting for 63.7 ± 3.8 ms for the left and 63.4 ± 3.1 ms for the right distal sciatic nerve. However, the T2 values detected by the 3D T2-prepared TSE sequence are in close agreement with the T2 values obtained by MRS that has been acquired in one volunteer and can be seen as the reference acquisition regarding T2 measurements.

A potential reason for the considerable discrepancy between the results derived from T2 mapping with the 2D MESE sequence and the 3D T2-prepared TSE sequence as well as MRS could be the influence of residual fat on the T2 quantification. Peripheral nerves are surrounded by fat and also partially consist of fat. The fat suppression technique of SPAIR does not suppress the olefinic fat peak and the residual fat signal could thus potentially lead to an overestimation of the pure nerve signal. Indeed, previous work in muscle tissue with fatty infiltration has shown that the employed 3D T2-prepared TSE sequence is less affected by the influence of fat and, thus, shows good agreement with the method of MRS for this purpose [43]. Other, minor factors contributing to discrepancies between T2 values of our investigation and the previous study might be represented by differences in placing the coil and defining the ROIs. However, previous research on other nerves of the PNS (e.g., tibial nerve or median nerve) among healthy subjects using different coils and ways of T2 extraction has also arrived at T2 values between 40 and 50 ms on average at 3 Tesla [44, 45]. Overall, this suggests that normal T2 values might be comparable between different nerves of the PNS in different body parts, with the chosen sequence probably being the most essential contributor to discrepancies when comparing values between studies.

So far, the focus of the small body of literature using (semi-)quantitative MRN for evaluations of the sciatic nerve was on systemic diseases with components affecting also the PNS or rather rarely observed diseases [15, 17, 18, 20, 21, 23–25]. However, other conditions, such as sciatica resulting from degenerative disc disease with nerve compression due to disc herniation, are much more common [29, 30]. Recently, MRN with T2 mapping has been shown to be able to detect elevated T2 values at the level of the LSP in compressed nerves when compared to the contralateral side or a control level without pathology, which was described even in the absence of clear qualitative, visually assessed signal alterations in affected nerves [41]. The present study extends these findings by showing more distant alterations of nerves by means of T2 values at the level of the distal sciatic nerve. Thus, while clinically used anatomical MRI at the LSP may detect structural changes and disc herniation, quantitative MRN using T2 mapping of the distal sciatic nerve might be capable of detecting direct nerve affection in a timely fashion. Follow-up studies may need to investigate further the alterations of T2 values in sciatica due to degenerative disc disease with disc herniation and unilateral nerve contact to explore how to best use this additional quantitative information. It seems possible that the magnitude of T2 alterations due to such conditions may show changes over time, which might even allow to discriminate between irreversibly affected nerves, e.g. in patients not responding well to later decompressive surgery, and nerves that are not lastingly affected.

As the study’s limitations, the size of the study cohort that was comparatively small has to be mentioned. Although forty-two T2 values derived from the 3D T2-prepared TSE sequence were obtained as a result of the segmentation of both the left-sided and right-sided distal sciatic nerves in healthy subjects, upcoming studies using data derived from larger cohorts are highly needed to provide normative data. Furthermore, factors such as sex, age, or body mass index should be considered in future studies as they may have an impact on T2 measurements. Moreover, we have only reported on a case series of patients, with upcoming studies having to confirm the results observed in the small number of patients of this investigation. However, the finding of differences in T2 values between sides in the patients and when comparing T2 values between healthy subjects and patients still points at the clinical relevance of quantitative MRN using T2 mapping. The multi-compartment signal decay of peripheral nerves should also be considered as a potential shortcoming regarding determination of T2 values, especially with respect to the desirable comparability of MRI sequences of different vendors or centers. In this study, a sequence with strong initial T2 weighting was employed that mitigates contributions of short T2 components on the signal decay curves. With the used 3D T2-prepared TSE sequence a very good agreement with MRS measurements was observed. This indicates that this sequence is well suited in the presented context but other sequences might have to be adapted to achieve a similar fat suppression and TE to deliver comparable results. Another limitation regarding the MRS measurements is the potential contribution of surrounding water-containing tissues. We tried to avoid muscle tissue surrounding the nerves when placing the VOI. However, contributions of the surrounding tissue due to partial volume averaging cannot be fully excluded but should be minor compared to the contribution of the nerves.

In conclusion, this is one of the first studies to apply quantitative MRN and to present T2 values of the distal sciatic nerve in healthy subjects. Our results suggest that T2 mapping derived from a 3D T2-prepared TSE sequence is highly reliable and, importantly, shows lower values than a commonly used 2D MESE sequence whilst being in agreement with the nerve water T2 from MRS. The influence of residual fat on the T2 quantification that is not entirely suppressed in a commonly used 2D MESE sequence may account for this discrepancy and should be considered during T2 mapping of the PNS. Furthermore, T2 mapping using a 3D T2-prepared TSE sequence might allow for evaluations of quantitative alterations of the distal sciatic nerve in patients with unilateral sciatica due to degenerative disc disease with unilateral nerve contact of degenerated disc material, which may allow T2 values of the distal sciatic nerve to become biomarkers of direct nerve affection in patients with degenerative changes at the level of the LSP.

## Abbreviations

2D: Two-dimensional
3D: Three-dimensional
a.u.: Arbitrary units
BMRC: British Medical Research Council
CIDP: Chronic inflammatory demyelinating polyneuropathy
CMTD: Charcot–Marie–Tooth disease
DTI: Diffusion tensor imaging
FOV: Field of view
LSP: Lumbosacral plexus
MRI: Magnetic resonance imaging
MRN: Magnetic resonance neurography
MRS: Magnetic resonance spectroscopy
MS: Multiple sclerosis
MESE: Multi-echo spin-echo
ODI: Oswestry Disability Index
PNS: Peripheral nervous system
R1: Reader 1
R2: Reader 2
RMSCV: Root mean square coefficient of variation
RMSE: Root mean square error
ROI: Region of interest
SPAIR: Spectral attenuated inversion recovery
STEAM: Stimulated echo acquisition mode
TE: Echo time
TM: Mixing time
TR: Repetition time
TSE: Turbo spin echo
VAS: Visual analogue scale
VOI: Volume of interest
